# Coordination-driven self-assembly vs dynamic covalent chemistry: versatile methods for the synthesis of molecular metallarectangles

**DOI:** 10.3762/bjoc.14.178

**Published:** 2018-08-03

**Authors:** Li-Li Ma, Jia-Qin Han, Wei-Guo Jia, Ying-Feng Han

**Affiliations:** 1 Key Laboratory of Synthetic and Natural Functional Molecule Chemistry, College of Chemistry and Materials Science, Northwest University, Xi’an 710127, China; 2 College of Chemistry and Materials Science, The Key Laboratory of Functional Molecular Solids, Ministry of Education, Anhui Normal University, Wuhu 241002, China

**Keywords:** coordination-driven self-assembly, dynamic covalent chemistry, half-sandwich rhodium complex, metallarectangles, one-pot reaction, supramolecular chemistry

## Abstract

Supramolecular coordination assemblies have a range of potential applications in chemical and biological sciences. Herein, simple modular methods for the synthesis of metallarectangles are described. The desired tetranuclear metallarectangles were synthesized by using coordination-driven self-assembly of half-sandwich rhodium-based organometallic clip units and organic ligands. The reaction of such an organometallic clip with 4-formylpyridine provided a dinuclear molecular tweezer with pendant aldehyde groups, and subsequent [4 + 4] condensation reactions with diamines provides another route to the target metallarectangles in good yields. The same assemblies can also be easily isolated in one-pot procedures by mixing the organometallic clip, diamines and 4-formylpyridine.

## Introduction

Over the past two decades, supramolecular structures with organometallic half-sandwich fragments have attracted much attention, including metallarectangles, metallacages and Borromean-type rings. Moreover, many of these structures have been utilized for various applications, such as catalysts, host–guest chemistry and others [1–17]. Through the use of a range of diverse functional ligands, the coordination-driven self-assembly has been proven to be a powerful tool to construct supramolecular architectures with controlled shapes and sizes [18–30]. Using this strategy, a host of exciting supramolecular structures have been constructed by using two elaborately designed building blocks, such as dinuclear half-sandwich molecular clips and appropriate pyridyl ligands. The sizes and structures of the obtained molecular rectangles, cages or rings can be easily tuned by adjusting the length and shape of the bridging ligands and molecular clips. We and others have reported a suite of [2 + 2] tetranuclear metallarectangles, each formed using dinuclear molecular clips and pyridyl-based donor ligands [6–9,31–36]. The introduction of dynamic covalent bonds (such as imine C=N bonds), could allow the multicomponent assembly of such architectures using rather simple precursors, however, studies along these lines are rare [37]. Thus, the preparation of single and discrete supramolecular architectures via dynamic covalent bond-driven self-assembly remains challenging. Severin and co-workers have recently shown that metallamacrocycles and cages based on half-sandwich ruthenium could be obtained in one-pot reactions from simple building blocks [38–39]. This finding prompted us to investigate whether condensation reactions between amines and 4-formylpyridine can be used simultaneously with coordination bond formation to construct metallarectangle structures in one-pot reactions, thereby reducing both waste and the number of reaction steps.

In this work we successfully combine coordination-driven self-assembly and dynamic covalent chemistry through imine bond formation between amines and 4-formylpyridine to construct the desired rectangular tetrarhodium molecular rectangles.

## Results and Discussion

**Scheme 1:**
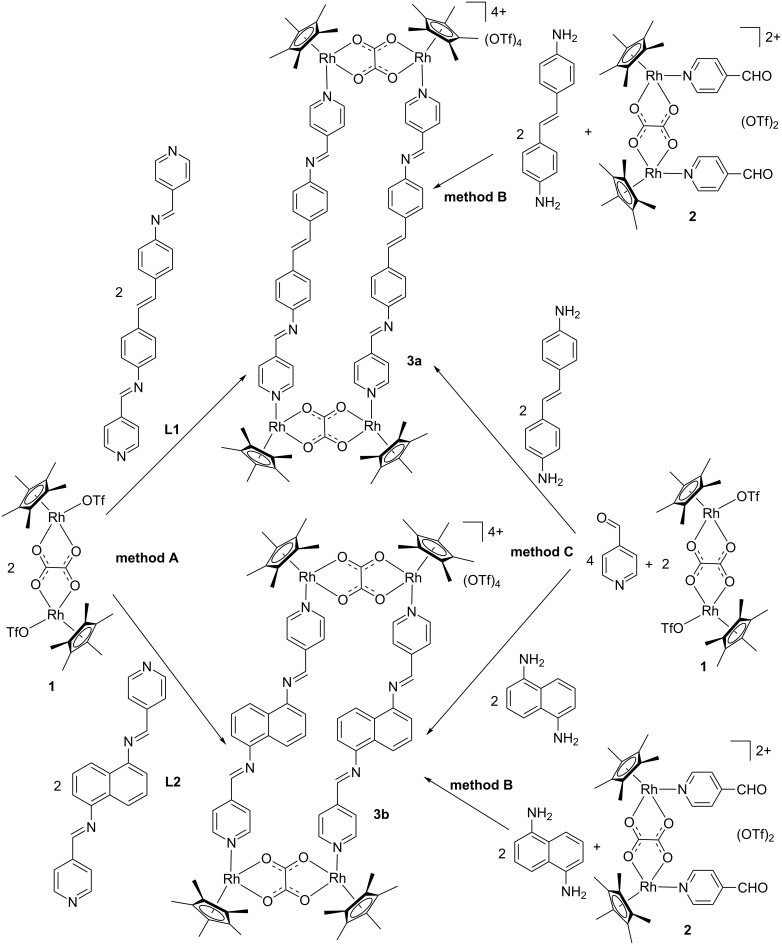
Synthesis of half-sandwich rhodium metallarectangles via three different methods. Method A: coordination-driven self-assembly of organometallic clips and organic ligands; method B: [4 + 4] condensation reactions of half-sandwich rhodium-based dialdehyde complexes with diamines; method C: assembly of metallarectangles with organometallic clip, diamines and 4-formylpyridine in a one-pot procedure.

The different approaches to the synthesis of tetranuclear molecular rectangles used in this work are shown in Scheme 1. We and others have used a two-step supramolecular design strategy for the formation of half-sandwich metal-based metallarectangles and metallacages [6–9]. Following this approach, two self-assembled metallarectangles with different bridging linkers **3a**,**b** were synthesized by utilizing the [Cp*_2_Rh_2_(μ-η^2^-η^2^-C_2_O_4_)Cl_2_] unit as molecular clips (Scheme 1, method A).

**Figure 1:**
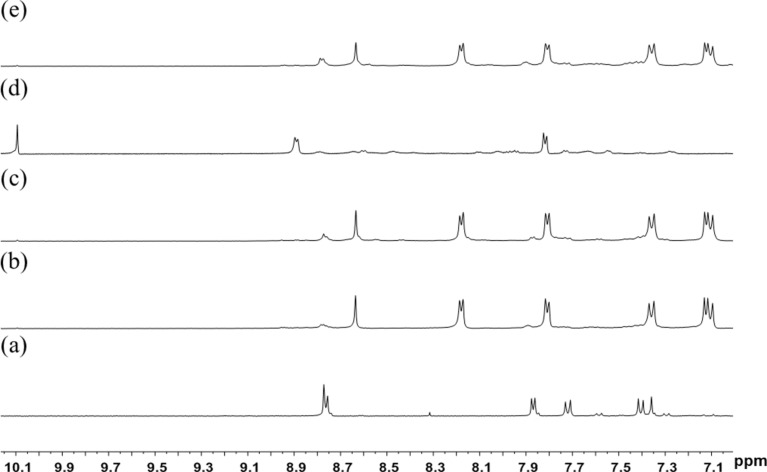
Partial ^1^H NMR spectra (400 MHz, DMSO-*d*_6_, ppm) of (a) **L1**; (b) the sample of metallarectagle **3a** obtained by coordination-driven self-assembly of organometallic clip **1** and **L1** (method A); (c) the sample of metallarectagle **3a** obtained through [4 + 4] condensation reactions of half-sandwich rhodium-based dialdehyde complex **2** with *trans*-4,4'-stilbenediamine (method B); (d) half-sandwich rhodium-based dialdehyde **2**; (e) the product of self-assembly of organometallic clip **1**, *trans*-4,4'-stilbenediamine and 4-formylpyridine in a one-pot procedure (method C).

Precursor complex **1**, which bears two labile triflato ligands was prepared in situ by chloride abstraction from [Cp*_2_Rh_2_(μ-η^2^-η^2^-C_2_O_4_)Cl_2_] with AgOTf. Stirring a mixture of **1** and **L1** in a 1:1 molar ratio in methanol for 24 h resulted in a homogeneous, dark-red solution. The ^1^H NMR spectrum of the obtained solution displays significant downfield shifts of the pyridyl signals, consistent with the loss of electron density upon coordination of the nitrogen atom to the metal centers (Figure 1a,b). Analysis of the reaction solution using electrospray ionization mass spectrometry (ESIMS) showed a signal at *m*/*z* = 476.1010, corresponding to a tertracation species of complex **3a**. The peak was isotopically resolved and agrees well with the theoretical isotopic distribution. In addition, the IR spectrum of the rhodium metallarectangle **3a** showed a C=N stretching band at 1618 cm^−1^.

**Figure 2:**
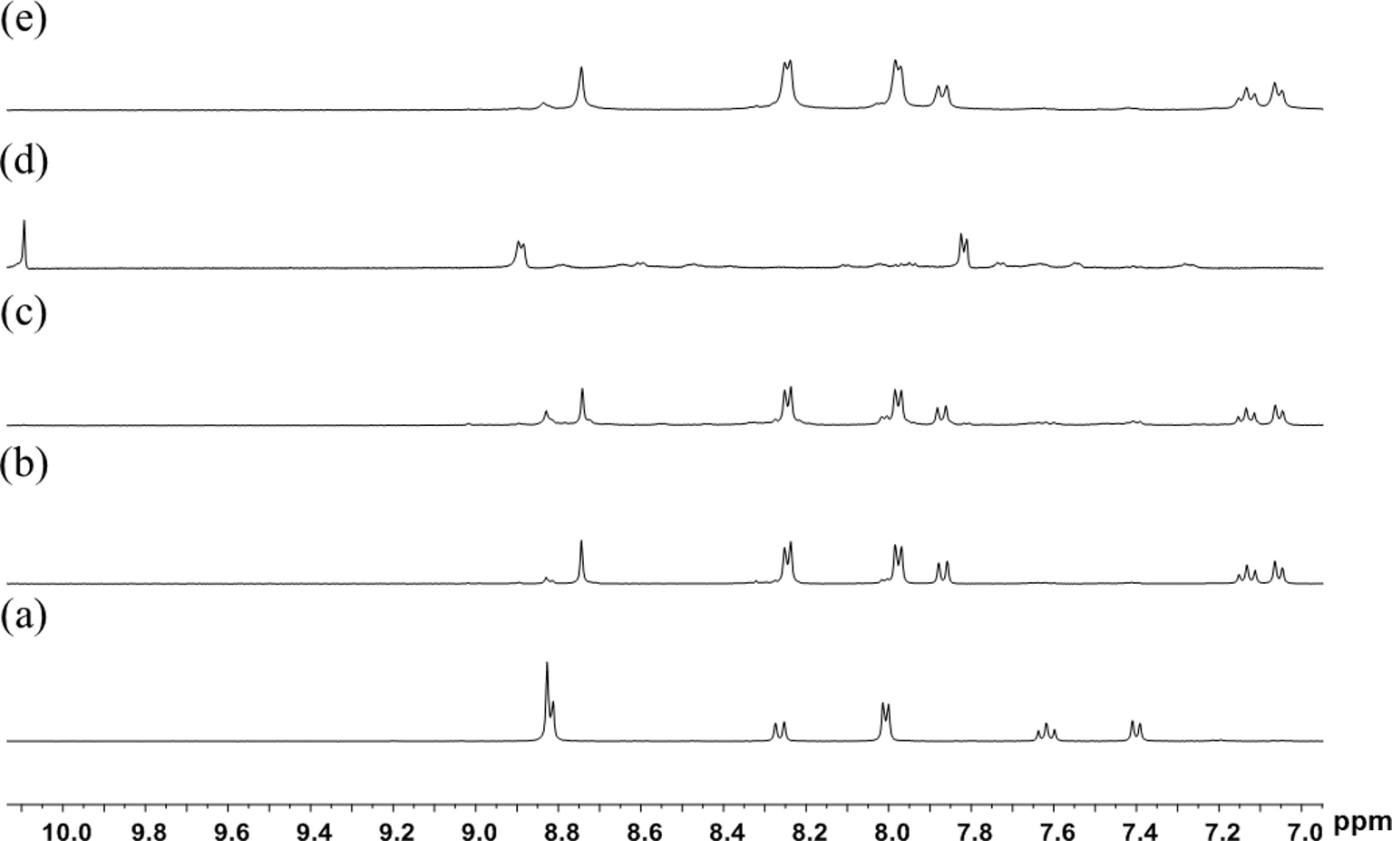
Partial ^1^H NMR spectra (400 MHz, DMSO-*d*_6_, ppm) of (a) **L2**; (b) a sample of metallarectangle **3b** obtained by coordination-driven self-assembly of organometallic clip **1** and **L2** (method A); (c) a sample of metallarectangle **3b** obtained by [4 + 4] condensation reactions of half-sandwich rhodium-based dialdehyde complex **2** with 1,5-diaminonaphthalene (method B); (d) half-sandwich rhodium-based dialdehyde **2**; (e) the product of assembly of organometallic clip **1**, 1,5-diaminonaphthalene and 4-formylpyridine in a one-pot procedure (method C).

**Figure 3:**
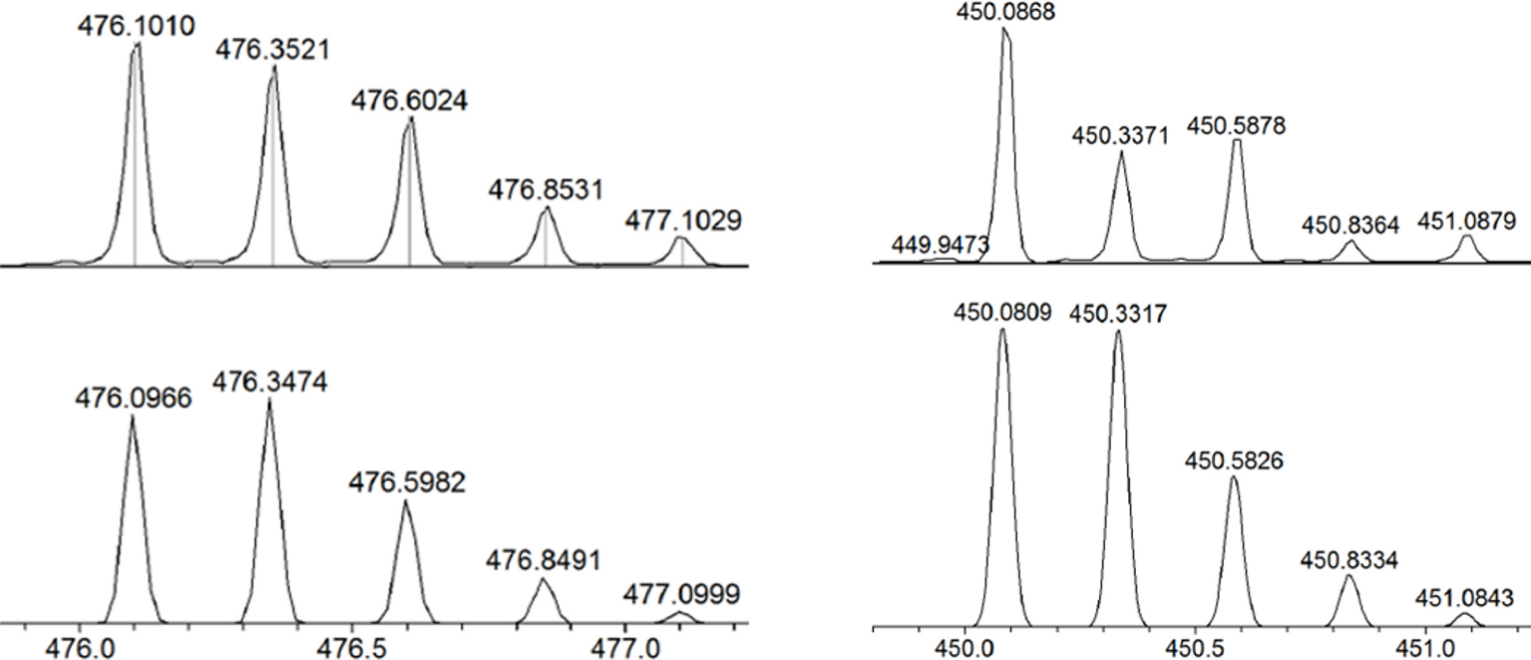
Calculated (bottom) and experimental (top) ESI-MS spectra of the tetracationic half-sandwich rhodium metallarectangles [**3a** – 4OTf]^4+^ (left) and [**3b** – 4OTf]^4+^ (right).

The same self-assembly protocol can also be used for the synthesis of metallarectangle **3b**. The combination of two labile-ligand precursor complexes **1** and two pyridyl-based ligands **L2** in a 1:1 molar ratio led to the formation of **3b** in good yield. The ^1^H NMR spectrum of the reaction mixture revealed the formation of a single species. In the ^1^H NMR spectrum of **3b**, only one sharp set of characteristic peaks was found. Again, significant downfield shifts of the pyridyl proton signals were observed, indicating the efficient self-assembly of the rhodium-based assembly (Figure 2a,b). Clear evidence for the formation of a discrete tetranuclear organometallic product was obtained from ESI mass spectrometry. Similar to that observed in complex **3a**, a peak at *m*/*z* = 450.0868 was observed, which is attributable to [**3b** − 4OTf]^4+^, and its isotopic pattern is in good agreement with the theoretical distribution (Figure 3, right). The absorption band at 1620 cm^−1^ in the IR spectrum indicated the existence of an imine group.

The geometries of the metallarectangles **3a** and **3b** were expected to be similar, as they comprise two oxalate-bridged half-sandwich rhodium fragments linked by two Schiff-base ligands **L1** or **L2**, giving the desired tetranuclear metallarectangles. In order to test the possibility of using dynamic covalent chemistry to assemble these metallarectangles, we attempted a further method (Scheme 1, method B) to synthesize these assemblies. As shown in Scheme 1, a dinuclear molecular tweezer complex **2** bearing two pendant aldehyde groups can be formed from the labile ligand complex **1** and 4-formylpyridine, and subsequent reaction with diamines would potentially give tetranuclear metallarectangles. When equimolar amounts of either *trans*-4,4'-stilbenediamine or 1,5-diaminonaphthalene were added to methanol solutions of complex **2**, and allowed to react for 24 h at room temperature, the formation of tetranuclear [4 + 4] condensation products **3a** (Figure 1c) and **3b** (Figure 2c) was observed, respectively. Complexes **3a** and **3b** were isolated in good yields, and their structures were confirmed by ^1^H NMR spectroscopy and ESI mass spectrometry.

After establishing that the condensation reaction of **2** with amines is an efficient method to form metallarectangles, we sought to test the possibility of forming the desired assemblies in a one-pot reaction, i.e., the combination of coordination-driven and dynamic covalent self-assembly strategies (Scheme 1, method C) [33]. When a mixture of the labile ligand complex **1**, *trans*-4,4'-stilbenediamine and 4-formylpyridine in a 1:1:1 molar ratio in methanol was allowed to react for 24 h at room temperature, the clear, quantitative formation of complex **3a** was revealed by NMR spectrometry (Figure 1e). The analogous one-pot construction of **3b** was also successful (Figure 2e). Notably, the isolated yields of the metallarectangles are higher than the overall yields of the two-step method.

**Figure 4:**
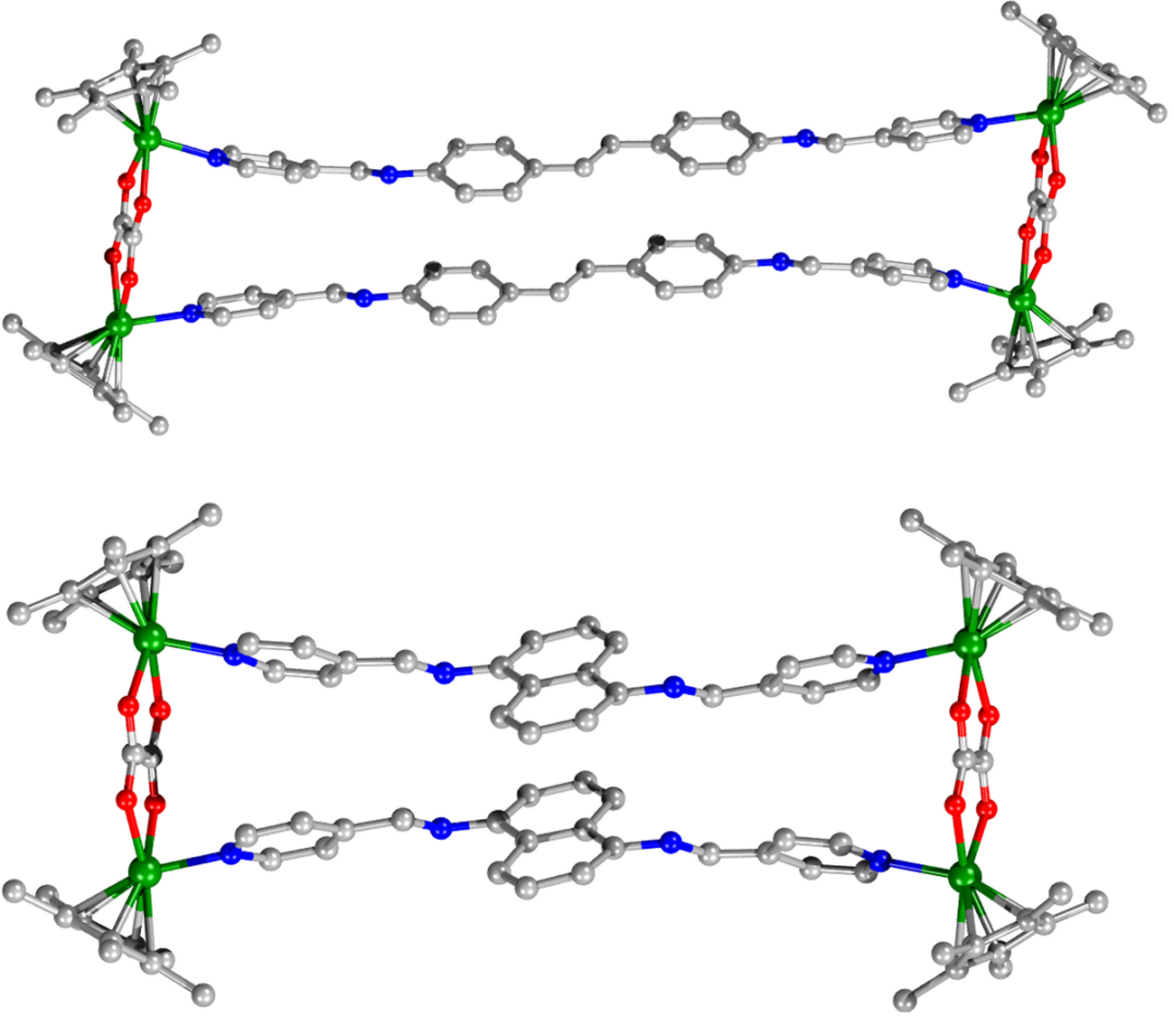
Optimized structures of the charged metallarectangles **3a** (top) and **3b** (bottom), optimized with the molecular mechanics force field. The graphics were produced using the Diamond software package. Colors: C, gray; O, red; N, blue; rhodium, green (hydrogen atoms have been removed for clarity).

Since attempts to obtain X-ray quality single crystals of the target metallarectangles were unsuccessful, molecular simulations were performed to gain further insight into the structures of the assemblies **3a** and **3b**. The optimized structures of each assembly featured a similar rectangular metallacyclic macrocycle structure (Figure 4). The sizes of the assembled structures were estimated to be 26.3 × 5.6 Å (**3a**) and 20.1 × 5.6 Å (**3b**).

## Conclusion

In summary, a modular protocol for the synthesis of metallarectangles is described. The desired tetranuclear metallarectangles can be obtained via three different approaches: 1) exploiting the coordination-driven self-assembly of half-sandwich rhodium-based organometallic clips and organic ligands, 2) [4 + 4] condensation reactions of diamines with dinuclear molecular tweezer complex bearing pendant aldehyde groups, and 3) a sample one-pot procedure involving mixing the organometallic clips, diamines and 4-formylpyridine. Our results thus present versatile and efficient approaches to the synthesis of molecular metallarectangles with intricate topologies. The methods shown here are potentially useful for the synthesis of functional molecular metallacages, and the experimental efforts in this direction are currently underway.

## Experimental

### Materials and methods

All manipulations were performed under an atmosphere of nitrogen using standard Schlenk techniques. Commercial grade solvents and reagents were used without further purification. [Cp*_2_Rh_2_(μ-η^2^-η^2^-C_2_O_4_)Cl_2_] [25], *trans*-4,4'-stilbenediamine [40] and **L2** [41] were prepared according to literature procedures. Methanol was purified by standard methods prior to use. NMR (400 MHz) spectra were obtained on a Bruker AVANCE III spectrometer. IR spectra of the solid samples (KBr tablets) were recorded on a Bruker EQUINOX-55 (TENSOR27) IR spectrometer. Mass spectra were obtained with UltiMate3000 spectrometers.

### General procedure for the synthesis of **L1** and **L2**

**L1**: To 4-formylpyridine (195 mg, 1.82 mmol) in dry CH_3_OH (20 mL) was added *trans*-4,4'-stilbenediamine (191 mg, 0.91 mmol) at room temperature. The mixture was stirred at room temperature for 24 h and then filtered. The resulting yellow solid was washed with methanol (2 × 5 mL) and diethyl ether (2 × 5 mL) to give **L1** (318 mg, 90%). ^1^H NMR (400 MHz, DMSO-*d*_6_, ppm) δ 8.77 (s, 2H, -NCH-), 8.76 (d, *J* = 6.0 Hz, 4H), 7.87 (d, *J* = 6.0 Hz, 4H), 7.72 (d, *J* = 8.4 Hz, 4H), 7.41 (d, *J* = 8.4 Hz, 4H), 7.36 (s, 2H, -CH=CH-); IR (KBr, cm^−1^): 3419 (m), 1597 (s), 1411 (m), 962 (m), 831 (s), 632 (m), 561 (s).

**L2**: A modified synthetic procedure adapted from literature methods [33] was used. To 4-formylpyridine (215 mg, 2.0 mmol) in dry CH_3_OH (20 mL) was added 1,5-diaminonaphthalene (160 mg, 1.0 mmol) at room temperature. The mixture was stirred at room temperature for 24 h and then filtered. The resulting yellow solid was washed with diethyl ether (2 × 3 mL) and crystallized from CH_2_Cl_2_/hexane (1:1) to give **L2** (220 mg, 65%). ^1^H NMR (400 MHz, DMSO-*d*_6_, ppm) δ 8.83 (s, 2H, -NCH-), 8.82 (d, *J* = 6.0 Hz, 4H), 8.26 (d, *J* = 8.4 Hz, 2H), 8.01 (d, *J* = 6.0 Hz, 4H), 7.62 (t, 2H), 7.40 (d, *J* = 7.2 Hz, 2H); IR (KBr, cm^−1^): 3024 (w), 1624 (s), 1597 (s), 1404 (s), 1317 (s), 1232 (s), 925 (s), 790 (s), 652 (m).

### Synthesis of complex **2** [Cp^*^_2_Rh_2_(μ-η^2^-η^2^-C_2_O_4_)(4-CHOPy)_2_](OTf)_2_

AgOTf (36 mg, 0.14 mmol) was added to a solution of [Cp^*^_2_Rh_2_(μ-η^2^-η^2^-C_2_O_4_)Cl_2_] (45 mg, 0.07 mmol) in CH_3_OH (15 mL) at room temperature and the mixture was stirred for 1 h, followed by filtration to remove insoluble materials. Then 4-formylpyridine (16 mg, 0.14 mmol) was added to the filtrate and the mixture was stirred for 24 h. The volume was reduced to 3 mL in vacuo. Upon the addition of diethyl ether, a light-yellow solid was precipitated and washed with diethyl ether (3 × 3 mL) and dried under vacuum (60 mg, 80%).^1^H NMR (400 MHz, DMSO-*d*_6_, ppm) δ 10.09 (s, 2H, -CHO), 8.89 (d, *J* = 5.0 Hz, 4H), 7.82 (d, *J* = 5.0 Hz, 4H), 1.55 (s, 30H, Cp*-H); HRMS–ESI (*m*/*z*): [**2** − 2OTf]^2+^ calcd for C_34_H_40_O_12_N_2_F_6_S_2_Rh_2_, 390.0571; found, 390.0541.

### Synthesis of [Cp*_4_Rh_4_(μ-η^2^-η^2^-C_2_O_4_)_2_(L1)_2_](OTf)_4_ (**3a**)

**Method A:** AgOTf (36 mg, 0.14 mmol) was added to a solution of [Cp^*^_2_Rh_2_(μ-η^2^-η^2^-C_2_O_4_)Cl_2_] (45 mg, 0.07 mmol) in CH_3_OH (30 mL) at room temperature and the mixture was stirred for 1 h, followed by filtration to remove insoluble materials. Then a solution of **L1** (27 mg, 0.07 mmol) in 15 mL CHCl_3_ was added dropwise to the filtrate. The mixture was stirred at room temperature for 24 h to give a deep red solution. The volume was reduced to 3 mL in vacuo. Upon the addition of diethyl ether, a black-red solid was precipitated and washed with CHCl_3_ (2 × 3 mL) and dried under vacuum (61 mg, 70%). ^1^H NMR (400 MHz, DMSO-*d*_6_, ppm) δ 8.64 (s, 4H, -NCH-), 8.18 (d, *J* = 6.0 Hz, 8H), 7.81 (d, *J* = 6.4 Hz, 8H), 7.36 (d, *J* = 8.8 Hz, 8H), 7.12 (d, *J* = 6.0 Hz, 8H), 7.09 (s, 4H), 1.57 (s, 60H, Cp*-H). HRMS–ESI (*m*/*z*): [**3a** − 4OTf]^4+^ calcd for C_100_H_100_O_20_N_8_F_12_S_4_Rh_4_, 476.0966; found, 476.0986.

**Method B: ***trans*-4,4'-Stilbenediamine (14 mg, 0.07 mmol) was added to a solution of **2** (76 mg, 0.07 mmol) in CH_3_OH (15 mL) at room temperature and the mixture was stirred for 24 h to give a deep red solution. Upon the addition of diethyl ether, a black-red solid was precipitated and washed with diethyl ether (3 × 3 mL) and dried under vacuum (63 mg, 72%). HRMS–ESI (*m*/*z*): [**3a** − 4OTf]^4+^ calcd for C_100_H_100_O_20_N_8_F_12_S_4_Rh_4_, 476.0966; found, 476.0963.

**Method C:** AgOTf (36 mg, 0.14 mmol) was added to a solution of [Cp^*^_2_Rh_2_(μ-*η*^2^-*η*^2^-C_2_O_4_)Cl_2_] (45 mg, 0.07 mmol) in CH_3_OH (10 mL) at room temperature and the mixture was stirred for 1 h, followed by filtration to remove insoluble materials. Then *trans*-4,4'-stilbenediamine (15 mg, 0.07 mmol) was added to the filtrate. A solution of 4-formylpyridine (15 mg, 0.14 mmol) in 7 mL CHCl_3_ was then added dropwise to the mixture and stirred for 24 h. The solvent was concentrated to about 3 mL. Diethyl ether was then added, and a black-red solid precipitated, which was washed with diethyl ether (3 × 3 mL) and chloroform (2 × 3 mL) and dried under vacuum (66 mg, 75%). HRMS–ESI (*m*/*z*): [**3a** − 4OTf]^4+^ calcd for C_100_H_100_O_20_N_8_F_12_S_4_Rh_4_, 476.0966; found, 476.1010.

### Synthesis of [Cp*_4_Rh_4_(μ-η^2^-η^2^-C_2_O_4_)_2_(L2)_2_](OTf)_4_ (**3b**)

**Method A:** AgOTf (36 mg, 0.14 mmol) was added to a solution of [Cp^*^_2_Rh_2_(μ-η^2^-η^2^-C_2_O_4_)Cl_2_] (45 mg, 0.07 mmol) in CH_3_OH (15 mL) at room temperature and the mixture was stirred for 1 h, followed by filtration to remove insoluble materials. Then a solution of **L2** (24 mg, 0.07 mmol) in 7 mL CHCl_3_ was added dropwise to the filtrate. The mixture was stirred at room temperature for 24 h and filtered. The resulting yellow solid was washed with chloroform (2 × 3 mL) and dried under vacuum (61 mg, 70%). ^1^H NMR (400 MHz, DMSO-*d*_6_, ppm) δ 8.74 (s, 4H, -NCH-), 8.25 (d, *J* = 6.0 Hz, 8H), 7.98 (d, *J* = 6.0 Hz, 8H), 7.87 (d, *J* = 8.8 Hz, 4H), 7.13 (t, 4H), 7.05 (d, *J* = 6.8 Hz, 4H), 1.59 (s, 60H, Cp*-H); HRMS–ESI (*m*/*z*): [**3b −** 4OTf]^4+^ calcd for C_92_H_92_O_20_N_8_F_12_S_4_Rh_4_, 450.0809; found, 450.0873.

**Method B:** 1,5-Diaminonaphthalene (11 mg, 0.07 mmol) was added to a solution of **2** (76 mg, 0.07 mmol) in CH_3_OH (20 mL) at room temperature and the mixture was stirred for 24 h to give a deep red solution. The volume was reduced to 3 mL in vacuo. Upon addition of diethyl ether, a yellow solid precipitated, which was washed with diethyl ether (3 × 5 mL) and dried under vacuum (61 mg, 73%). HRMS–ESI (*m*/*z*): [**3b** − 4OTf]^4+^ calcd for C_92_H_92_O_20_N_8_F_12_S_4_Rh_4_, 450.0809; found, 450.0807.

**Method C:** AgOTf (36 mg, 0.14 mmol) was added to a solution of [Cp^*^_2_Rh_2_(μ-η^2^-η^2^-C_2_O_4_)Cl_2_] (45 mg, 0.07 mmol) in CH_3_OH (15 mL) at room temperature and the mixture was stirred for 1 h, followed by filtration to remove insoluble materials. Then, 1,5-diaminonaphthalene (11 mg, 0.07mmol) was added to the filtrate. A solution of 4-formylpyridine (15 mg, 0.14 mmol) in 7 mL CHCl_3_ was added dropwise to the mixture and stirred for 24 h. A yellow solid precipitated, which was washed with chloroform (2 × 3 mL) and dried under vacuum (60 mg, 72%). HRMS–ESI (*m*/*z*): [**3b** − 4OTf]^4+^ calcd for C_92_H_92_O_20_N_8_F_12_S_4_Rh_4_, 450.0809*;* found, 450.0868.
